# Mortality Prediction of COVID-19 Patients at Intensive Care Unit Admission

**DOI:** 10.7759/cureus.19690

**Published:** 2021-11-18

**Authors:** Rajarajan Ganesan, Varun Mahajan, Karan Singla, Sushant Konar, Tanvir Samra, Senthil K Sundaram, Vikas Suri, Mandeep Garg, Naveen Kalra, Goverdhan D Puri

**Affiliations:** 1 Anaesthesia and Intensive Care, Postgraduate Institute of Medical Education and Research, Chandigarh, IND; 2 Internal Medicine, Postgraduate Institute of Medical Education and Research, Chandigarh, IND; 3 Radiodiagnosis, Postgraduate Institute of Medical Education and Research, Chandigarh, IND

**Keywords:** covid-19 ards, acute respiratory distress syndrome [ards], severe covid-19, mortality prediction, mortality, covid-19

## Abstract

Background

Coronavirus-2019 (COVID-19) patients admitted to the intensive care unit (ICU) have mortality rates between 30%-50%. Identifying patient factors associated with mortality can help identify critical patients early and treat them accordingly.

Patients and methods

In this retrospective study, the records of patients admitted to the COVID-19 ICU in a single tertiary care hospital from April 2020 to September 2020 were analysed. The clinical and laboratory parameters between patients who were discharged from the hospital (survival cohort) and those who died in the hospital (mortality cohort) were compared. A multivariate logistic regression model was constructed to identify parameters associated with mortality.

Results

A total of 147 patients were included in the study. The age of the patients was 55 (45, 64), median (IQR), years. At admission, 23 (16%) patients were on mechanical ventilation and 73 (50%) were on non-invasive ventilation. Sixty patients (40%, 95% CI: 32.8 to 49.2%) had died. Patients who died had a higher Charlson comorbidity index (CCI): 3 (2, 4) vs. 2 (1, 3), p = 0.0019, and a higher admission sequential organ failure assessment (SOFA) score: 5 (4, 7) vs. 4 (3, 4), p < 0.001. Serum urea, serum creatinine, neutrophils on differential leukocyte count, neutrophil to lymphocyte ratio (N/L ratio), D-dimer, serum lactate dehydrogenase (LDH), and C-reactive protein were higher in the mortality cohort. The ratio of partial pressure of arterial oxygen to fraction of inspired oxygen, platelet count, lymphocytes on differential leukocyte count, and absolute lymphocyte count was lower in the mortality cohort. The parameters and cut-off values used for the multivariate logistic regression model included CCI > 2, SOFA score > 4, D-dimer > 1346 ng/mL, LDH > 514 U/L and N/L ratio > 27. The final model had an area under the curve of 0.876 (95% CI: 0.812 to 0.925), p < 0.001 with an accuracy of 78%. All five parameters were found to be independently associated with mortality.

Conclusions

CCI, SOFA score, D-dimer, LDH, and N/L ratio are independently associated with mortality. A model incorporating the combination of these clinical and laboratory parameters at admission can predict COVID-19 ICU mortality with good accuracy.

## Introduction

The coronavirus disease 2019 (COVID-19) pandemic is caused by the severe acute respiratory syndrome coronavirus 2 (SARS-CoV-2). The disease spectrum extends from asymptomatic infection to severe disease and death [1]. Optimum resource utilization is of paramount importance as the multitude of cases has overburdened the healthcare system. Various observational studies have attempted to identify risk factors and test predictive models for mortality in COVID-19 patients [2,3]. Advanced age, male sex, presence of comorbid illnesses, higher sequential organ failure assessment (SOFA) score, raised D-dimer, ferritin, neutrophil counts, and lymphopenia are a few predictors for the early identification of critically ill patients.

Our present study aimed to study the clinical and laboratory parameters associated with mortality in COVID-19 patients admitted to the intensive care unit (ICU) and to create a mortality prediction model with logistic regression.

## Materials and methods

This retrospective observational study was conducted with the approval of the institutional ethics committee at the Postgraduate Institute of Medical Education and Research, Chandigarh, India (reference no. NK/6626/Study/032). The study included patients older than 18 years of age with COVID-19 pneumonia confirmed by reverse transcriptase-polymerase chain reaction (RT-PCR) for SARS-CoV-2, admitted to the intensive care unit (ICU) from April till September 2020 at Postgraduate Institute of Medical Education and Research, Chandigarh, India. Patients with severe COVID-19 pneumonia as defined by the presence of dyspnea, a room air saturation of less than 94% on room air, ratio of partial pressure of oxygen to fraction of inspired oxygen (P/F ratio) less than 300 mmHg, respiratory rate of more than 30 breaths/minute or lung infiltrates more than 50% on radiological examination, were eligible for admission to the ICU.

Exclusion criteria for the study included pregnant patients, patients receiving palliative care, post-operative patients, and patients with impaired oxygenation due to pulmonary edema, which could be fully explained by cardiac failure or volume overload.

The study data collected from the patient record at admission included patient age, comorbidities, Charlson comorbidity index (CCI) calculated from age and comorbidities [4], mode of ventilation, neurological function, hemodynamic status, inotropic requirement, blood gas analysis value of arterial partial pressure of oxygen and P/F ratio. Apart from these, the results of hematological and biochemical investigations obtained at the time of ICU admission were also extracted. These data were used to calculate the admission sequential organ failure assessment (SOFA) score [5]. The primary outcome was hospital mortality (mortality) or discharge (survival). Outcome data were collected from the hospital information system. The baseline characteristics (demographics, comorbid illness), SOFA score, and laboratory parameters were compared between survival and mortality. The study complies with the Strengthening the Reporting of Observational Studies in Epidemiology (STROBE) guidelines. 

Patients were managed as per the institutional protocol. Specifically, the treatment included steroids, anticoagulants, immunomodulators, and antiviral agents. Lung protective ventilation strategy was used for all patients [6], and prone positioning was a part of management in all patients.

Statistical analysis

Categorical data were analyzed with the Pearson Chi-square test or the Fisher’s exact test for difference between survival and mortality cohorts. Continuous variables were compared using the Mann-Whitney U test, with a p value of < 0.05 being considered as significant. The descriptive data are presented as the median and interquartile range (IQR). Univariate logistic regression for mortality was tested on those parameters which were available for all patients, and which were found to be significantly different on the Mann-Whitney U test. A correlation matrix was plotted to exclude the parameters demonstrating collinearity. Receiver operator characteristic (ROC) curves were constructed to determine the cut-off values, which were used for dichotomizing the data for multivariate logistic regression. Statistical analysis was performed using Medcalc Statistical Software version 19.2.6 (MedCalc Software Ltd, Ostend, Belgium). 

## Results

**Figure 1:**
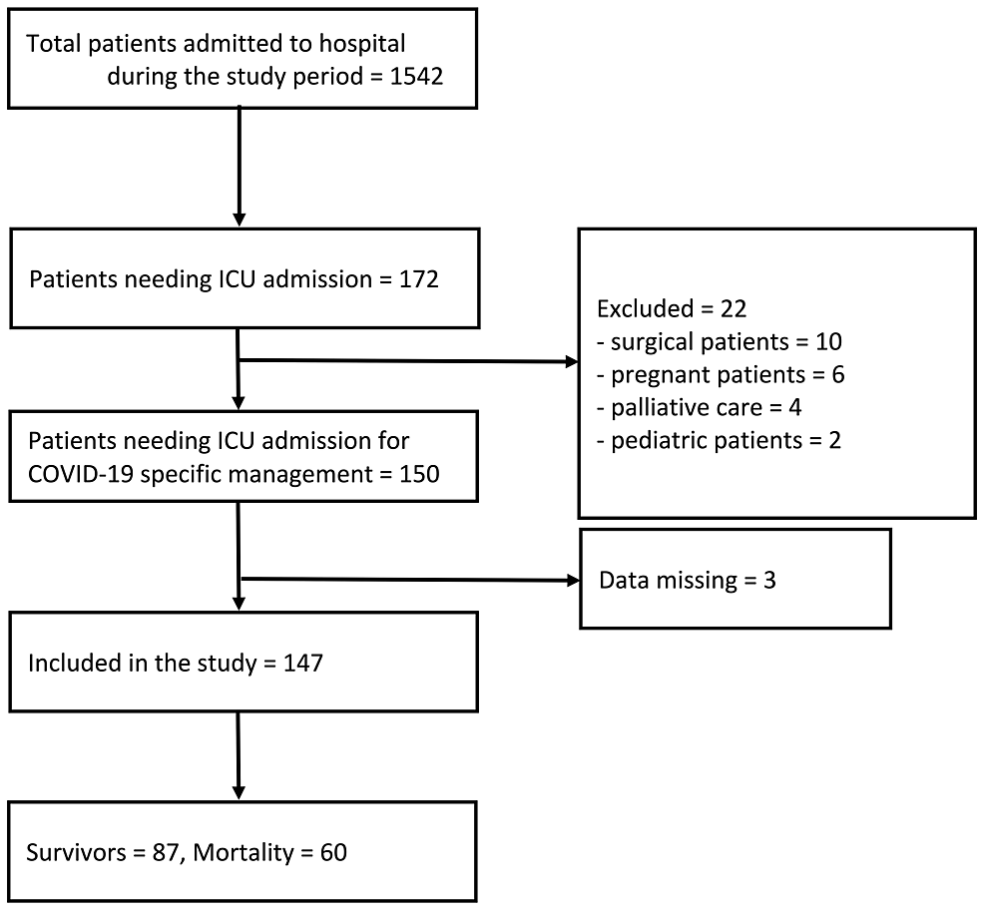
Study flow diagram. Abbreviations: COVID-19: coronavirus disease 2019, ICU: intensive care unit.

A total of 1542 patients were admitted to the hospital during the study period, and data from 147 eligible patients were analysed. Among the study patients, 87 (59.2%) survived (survival cohort), and 60 (40.8%) died (mortality cohort) (Figure 1).

Table 1 depicts the demographic characteristics and the oxygen support at admission in the overall study population, survival, and mortality cohorts.

**Table 1:** Demographic characteristics and oxygen device at admission. Non-parametric data presented as median (IQR) and compared between the two groups using Mann-Whitney U test. Nominal data presented as absolute number (percentage) and analysed using Pearson's Chi-square test. The percentages in brackets indicate value within the group. The p values in bold are significant (<0.05). ^a^ Includes pulmonary tuberculosis, chronic obstructive pulmonary disease, and bronchial asthma. ^b ^Low flow oxygen includes non-rebreather mask, Venturi mask and nasal prongs. ICU: intensive care unit.

Parameter	Overall (n = 147)	Survival (n = 87)	Mortality (n = 60)	p value
Demographics				
Age (in years)	55 (45, 64)	54 (43, 61)	58 (49, 67)	0.057
Sex (Males)	95	57 (65.5%)	38 (63%)	0.785
Hypertension	67	35 (40%)	32 (53%)	0.117
Diabetes mellitus	63	38 (43.6%)	25 (42%)	0.809
Coronary artery disease	17	7 (8%)	10 (16.7%)	0.108
Obesity (Body mass index>30 kg/m^2^)	34	16 (18.4%)	18 (30%)	0.101
Chronic kidney disease	13	3 (3.4%)	10 (16.7%)	0.006
Hypothyroidism	11	8 (9%)	3 (5%)	0.342
Pre-existing pulmonary disease^a^	11	7 (8%)	4 (6.7%)	0.755
Oxygen device at admission				
Invasive mechanical ventilation	23	4 (4.5%)	19 (32%)	<0.001
Non-invasive ventilation/ High flow nasal cannula	73	39 (44.8%)	34 (56.7%)	0.158
Low flow oxygen^b^	51	44 (50.6%)	7 (11.7%)	<0.001

There was no difference in age between the survival and mortality cohorts. There was a preponderance of male patients in the study (n=95, 64.6%). However, the sex distribution was not different between the two cohorts. Hypertension (n=67, 45%) and diabetes mellitus (n=63, 42%) were the most prevalent comorbid illnesses. Chronic kidney disease was more prevalent in patients who died (n = 10, 77% vs. n = 3, 23%; p = 0.006 in the mortality and survivor cohorts, respectively). At admission, 23 patients (16%) were on invasive mechanical ventilation, 73 (50%) were on non-invasive ventilation or high flow nasal oxygen and 51 (34%) were on low flow oxygen. Patients who died had a longer ICU stay, 10 (6, 14) vs. 7 (4, 11) days, p = 0.005.

**Figure 2:**
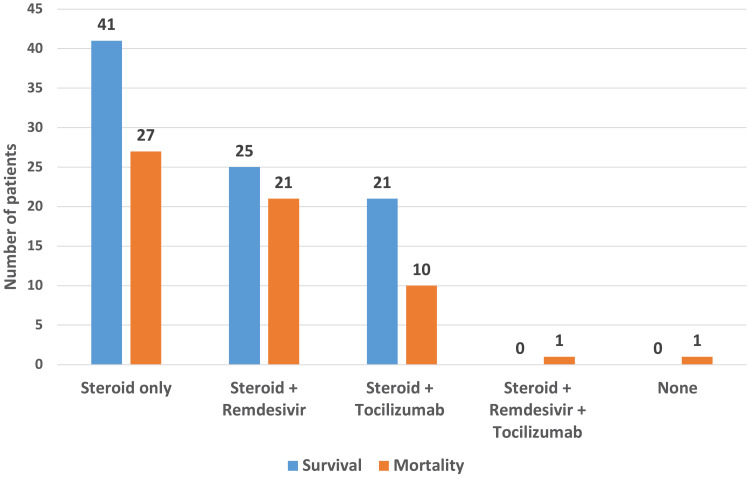
COVID-19 specific drug therapy. Steroid (injection methylprednisolone) was administered as an intravenous dose of 40 mg twice a day for 10 days (prolonged if persistent hypoxia), remdesivir as 200 mg on day one followed by 100 mg intravenous once a day for four days, and tocilizumab at a dose of 8 mg/kg intravenous.

Figure 2 presents the patient outcome in the context of the COVID-19 specific medical management that was administered.

**Figure 3:**
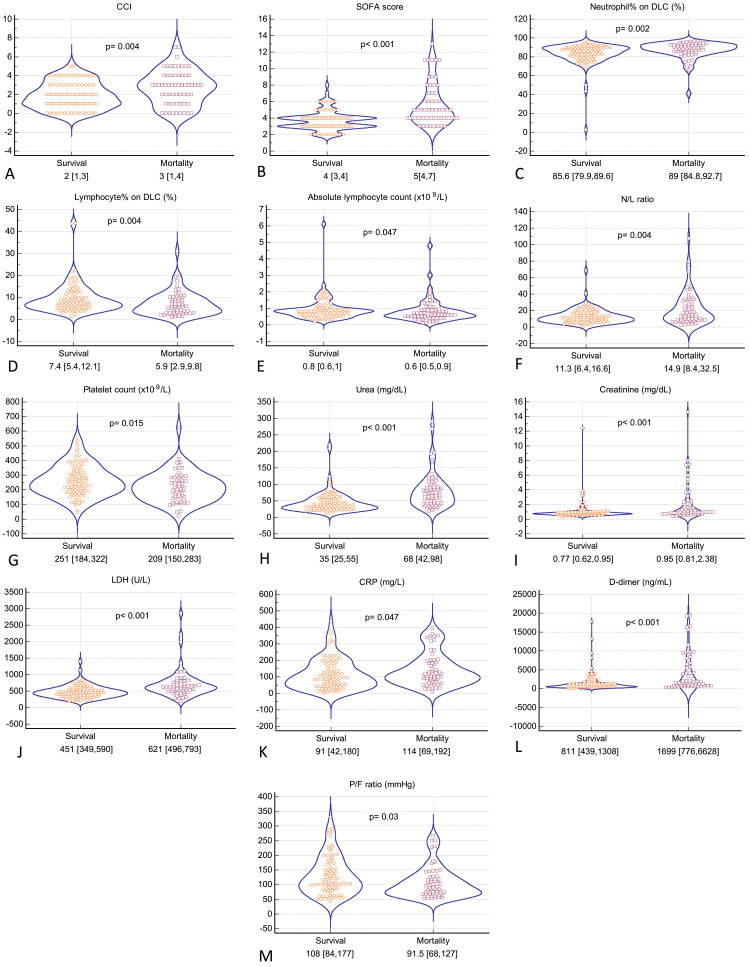
Violin plots with dots (A-M) for clinical and laboratory parameters which were different in the survival and mortality groups. Values presented on x-axis of each graph are the median (IQR) in the two groups. The p values mentioned in the graphs were obtained by Mann-Whitney U test. CCI: Charlson comorbidity index, CRP: C-reactive protein, DLC: differential leucocyte count, LDH: lactate dehydrogenase, P/F ratio: ratio of partial pressure of arterial oxygen and fraction of inspired oxygen, SOFA: sequential organ failure assessment score.

Figure 3 depicts the admission parameters, which were significantly different between the two groups.

The SOFA score, CCI, serum urea, serum creatinine, neutrophils on differential leukocyte count (DLC), neutrophil to lymphocyte ratio (N/L ratio), D-dimer, serum lactate dehydrogenase (LDH), and CRP were higher in the mortality cohort (Figure 3). The P/F ratio, platelet count, lymphocytes on DLC, and absolute lymphocyte count were lower in the mortality cohort. Table 2 depicts the comparison of all other laboratory parameters which were not different between the two cohorts.

**Table 2:** Laboratory parameters at admission which were not different between the two groups. Non-parametric data presented as median (IQR) and compared between the two groups using Mann-Whitney U test. ALP: alkaline phosphatase, ALT: alanine aminotransferase, AST: aspartate aminotransferase, TLC: total leucocyte count.

Parameter	Overall (n = 147)	Survival (n = 87)	Mortality (n = 60)	p value
Sodium (mmol/L)	139 (134, 143)	138 (134, 143)	139 (135.4, 143.6)	0.601
Potassium (mmol/L)	4.3 (3.8, 4.7)	4.2 (3.7, 4.68)	4.4 (3.8, 4.8)	0.234
Protein (g/dL)	6.5 (6.06, 7.03)	6.5 (6, 6.9)	6.5 (6, 7.14)	0.836
Albumin (g/dL)	3.24 (2.98, 3.51)	3.28 (3, 3.56)	3.2 (2.9, 3.4)	0.064
AST (U/L)	48.35 (33.5, 76.4)	46.5 (36, 76.8)	58 (32.4, 79)	0.330
ALT (U/L)	44.1 (29.3, 73.8)	46.4 (30, 67)	40.45 (27.2, 76.2)	0.604
ALP (U/L)	94(71, 132)	98 (70, 130)	88.5 (71.5, 149)	0.771
Bilirubin (mg/dL)	0.48 (0.38, 0.68)	0.47 (0.37, 0.63)	0.52 (0.39, 0.72)	0.594
Hemoglobin (gm/dL)	11.8 (10.45, 12.7)	11.8 (10.8, 12.65)	11.9 (9.85, 12.8)	0.905
TLC (x 10^9^/L)	11.6 (8, 15.05)	11.1 (8.15, 14.05)	12.15 (8, 16.12)	0.073

In addition, serum creatine kinase-MB (CK-MB), ferritin, troponin T, pro-brain natriuretic peptide (pro-BNP), and procalcitonin were available only in a subset of the study population, and these were significantly higher in the mortality cohort (Table 3).

**Table 3:** Biochemical parameters at admission which were available for a subset of patients. Data presented as median (IQR) and compared between the two groups using Mann-Whitney U test. The p values in bold are significant (<0.05). CKMB: creatine kinase-MB, pro-BNP: pro-brain type natriuretic peptide, PCT: procalcitonin.

Parameter	Overall	Survival	Mortality	p value
CKMB (U/L), n = 133	29.7 (22.35, 40.75)	27.3 (20.7, 37)	30.85 (24.8, 47.9)	0.042
Ferritin (ng/mL), n = 132	891 (405, 1381)	676 (337, 1160)	1080 (611, 1701)	<0.001
Troponin-T (pg/mL), n = 120	14.5 (7.14, 45)	10.5 (5.2, 22.8)	26.4 (9.7, 90)	<0.001
ProBNP (pg/mL), n = 128	538 (199.5, 2163)	164.5 (164, 740.9)	1680 (355, 3659)	<0.001
PCT (ng/mL), n = 101	0.288 (0.088, 1.003)	0.169 (0.05, 0.331)	0.675 (0.239, 4.05)	0.013

On univariate regression, CCI, SOFA score, P/F ratio, serum urea, serum creatinine, platelet count, neutrophils and lymphocyte on DLC, N/L ratio, D-dimer, and LDH were associated with mortality (Table 4).

**Table 4:** Univariate analysis for parameters identified as different between the two groups in Mann-Whitney U test. *Parameters used for the multivariate model. The p values in bold are significant (<0.05). CCI: Charlson’s comorbidity index, CRP: C-reactive protein, LDH: lactate dehydrogenase, N/L ratio: neutrophil to lymphocyte ratio, PCT: procalcitonin, P/F ratio: ratio of partial pressure of arterial oxygen and fraction of oxygen, SOFA: sequential organ function assessment.

Parameters	Odds ratio	95% CI	p value	AUC	95% CI
SOFA score*	1.9203	1.4478 - 2.5470	<0.0001	0.759	0.681 - 0.825
CCI*	1.4447	1.1530 - 1.8101	0.0008	0.648	0.565 - 0.725
P/F Ratio (mmHg)	0.9934	0.9874 - 0.9993	0.0284	0.605	0.522 - 0.685
Urea (mg/dL)	1.0230	1.0113 - 1.0349	<0.0001	0.762	0.685 - 0.828
Creatinine (mg/dL)	1.4758	1.1141 - 1.9551	0.0005	0.700	0.619 - 0.772
Platelet count (x 10^9^/L)	0.9961	0.9927 - 0.9994	0.0171	0.618	0.534 - 0.696
Neutrophil%	1.0905	10.0208 - 1.1649	0.0070	0.590	0.503 - 0.672
Lymphocyte%	0.9295	0.8696 - 0.9935	0.0104	0.640	0.557 - 0.717
N/L ratio*	1.0506	1.0196 - 1.0825	0.0001	0.641	0.558 - 0.719
Absolute lymphocyte count (x 10^9^/L)	0.7840	0.5030 - 1.2222	0.1830	0.599	0.513 - 0.681
D-dimer* (ng/mL)	1.0002	1.0001 - 1.0003	<0.0001	0.715	0.633 - 0.787
PCT (ng/mL)	1.0288	0.9979 - 1.0606	0.0191	0.750	0.654 - 0.831
LDH* (U/L)	1.0034	1.0016 - 1.0052	<0.0001	0.729	0.649 - 0.800
CRP (mg/L)	1.0031	0.9996 - 1.0066	0.0774	0.597	0.513 - 0.677

**Figure 4:**
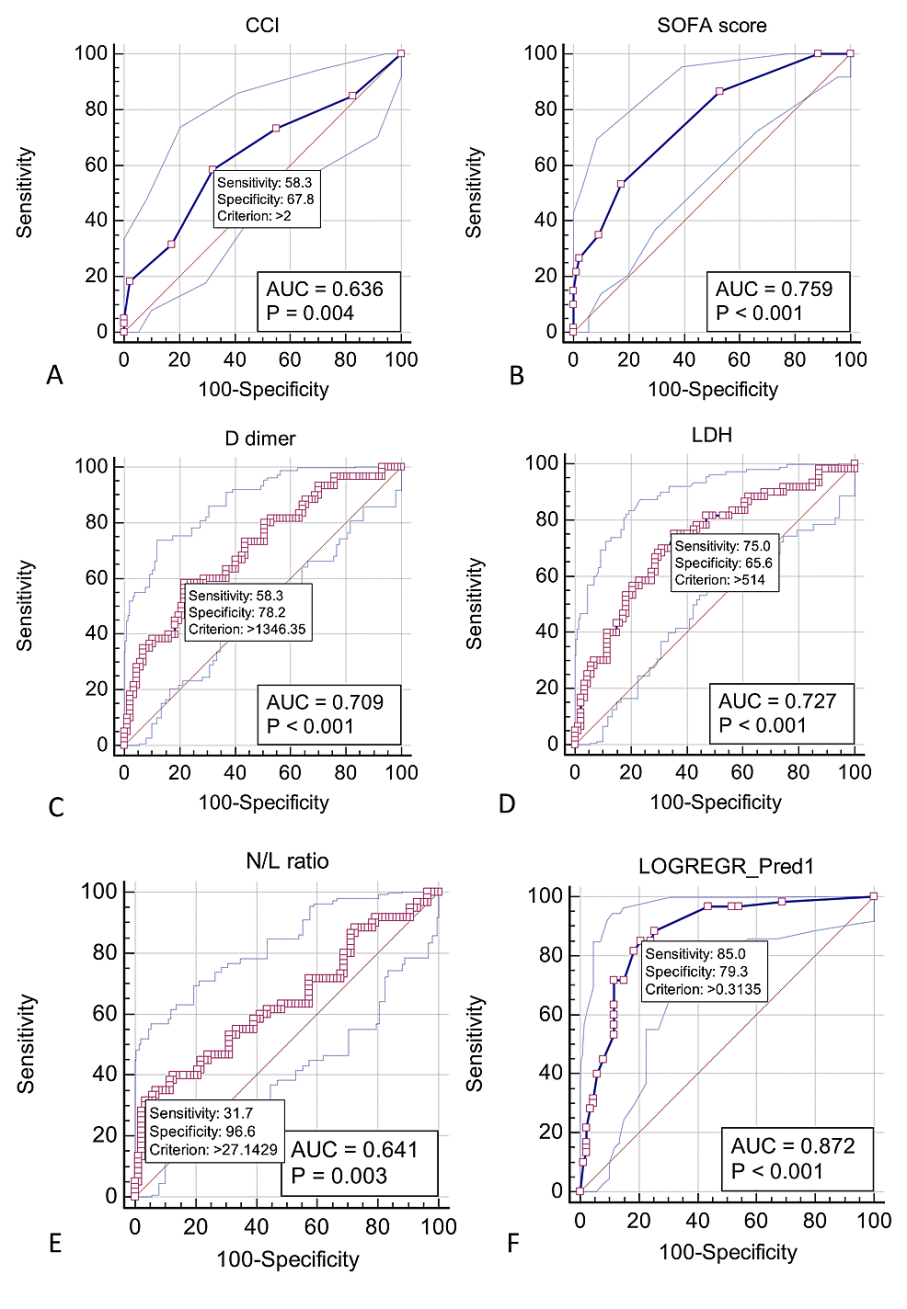
Receiver operator characteristics curves to obtain cut-off values for parameters used in the multivariate model. A-E presents the ROC curves for the parameters used in the multivariate model. F presents the ROC curve for the multivariate model. AUC: area under the curve, CCI: Charlson comorbidity index, LDH: lactate dehydrogenase, N/L ratio: neutrophil to lymphocyte ratio.

For multivariate logistic regression, SOFA score, which includes scores for P/F ratio and serum creatinine, and N/L ratio, which includes the percentage of neutrophil and lymphocytes in DLC, were utilized in place of the respective individual values. The area under the curve (AUC) (95% CI) for prediction of mortality for CCI, SOFA score, D-dimer, LDH and N/L ratio were 0.648 (0.565 to 0.725), p= 0.001; 0.759 (0.681 to 0.825), p < 0.001; 0.709 (0.629 to 0.781), p < 0.001; 0.727 (0.647 to 0.797), p < 0.001; and 0.641 (0.558 to 0.719), p = 0.003, respectively (Figure 4).

The cut-off values used for dichotomising the variables for multivariate regression were a CCI > 2 (sensitivity 61.7% and specificity 65.5%), SOFA score > 4 (sensitivity 53.3% and specificity 82.8%), D-dimer > 1346 ng/mL (sensitivity 58.3% and specificity 78.2%), LDH > 514 U/L (sensitivity 75% and specificity 65.6%) and N/L ratio > 27 (sensitivity 31.7% and specificity 96.6%). These five parameters (CCI, SOFA score, N/L ratio, D-dimer and LDH) were tested for association with mortality using multivariate logistic regression and were all found to be independently associated with mortality (Table 5).

**Table 5:** Multivariate logistic regression. Area under the ROC curve (95% CI) for the model is 0.876 (0.812 - 0.925), p < 0.0001. aOR: adjusted odds ratio, CCI: Charlson comorbidity index, LDH: lactate dehydrogenase, N/L ratio: neutrophil to lymphocyte ratio. The p values in bold are significant (< 0.05).

Parameter	Cut-off	aOR	95% CI for aOR	p value
CCI	> 2	2.6897	1.1505 - 6.2883	0.0224
SOFA score	> 4	2.6287	1.0719 - 6.446	0.0347
D-dimer (ng/mL)	> 1346.35	2.8391	1.1656 - 6.9153	0.0216
LDH (U/L)	> 514	5.4853	2.2696 - 13.2569	0.0002
N/L ratio	> 27.14	8.2153	1.8851 - 35.8024	0.0050

The final model was significant with an AUC (95% CI) of 0.876 (0.812 to 0.925), p < 0.0001 with an accuracy of 78%.

## Discussion

Our study in critically ill COVID-19 patients identified D-dimer, lactate dehydrogenase, and neutrophil to lymphocyte ratio to be associated with mortality. The association of these parameters with mortality was independent of each other as well as that of the commonly used CCI and admission SOFA score. Application of this evidence in clinical practice will help the physicians in early identification of critical patients and treat accordingly. 

Older age and comorbidities have earlier been shown to be associated with in-hospital and intensive care mortality in COVID-19 patients [7,8]. To account for the cumulative effect of age and comorbidities, we utilized CCI for analysis [4]. The cutoff value of > 2 for CCI identified in our study correlates with the finding of a systematic review in COVID-19 [9]. Moreover, there was a higher number of chronic kidney disease patients who died (77% vs. 23%). Chronic kidney disease is well recognized to cause severe COVID-19 and is an independent risk factor for mortality [10]. SOFA score has variably shown to predict mortality in COVID-19 patients. While one study had shown an admission SOFA score ≥ 3 to be predictive of in-hospital mortality (AUC: 0.890), another study on SOFA score, calculated 48 hours before mechanical ventilation, did not show adequate accuracy (AUC: 0.590) [11,12]. The accuracy of admission SOFA score in our ICU patient population intersects these two studies (AUC: 0.759) with a cut-off value of > 4.

Elevated D-dimer levels in COVID-19 due to hypercoagulability could be a key indicator of mortality in COVID-19 [13]. Various thresholds for D-dimer values have been proposed, with most of the values lying between 1000 and 2500 ng/mL [14-16]. However, we observed more sensitivity at values below 1500 ng/mL, and the Youden index was maximum at 1346 ng/mL. Lactate dehydrogenase can be elevated in COVID-19 as a result of multiple organ injuries [17]. Again, various cutoff values have been studied, ranging from 250 to 500 U/L. We obtained sensitivity and specificity of around 70% each for elevated LDH in predicting mortality. Again, this has been observed elsewhere [18]. Neutrophilia is observed in COVID-19 either due to inflammation or steroid use [19]. Coexisting lymphopenia results in an increase in the ratio of neutrophils to lymphocytes. The resulting increase in the N/L ratio has been previously shown to have a hazard ratio of around 1.05 for in-hospital mortality, as has also been observed in our study [20]. However, our cutoff value of 27 was chosen according to the Youden index, and it has a high specificity (96.6%).

Various studies have attempted to build models for predicting COVID-19 mortality [2,21,22]. The strengths of our study include the inclusion of only critically ill patients and the selection of parameters for testing. We have included the baseline risk status of the patients in the form of CCI and the clinical condition of the patient in the form of SOFA score. To add further granularity, the laboratory parameters were included. The laboratory parameters which we observed to be independently associated with mortality also appear to have a scientific basis. We could hypothesize that while D-dimer (endotheliitis) and neutrophil to lymphocyte ratio (dysregulated immune response) indicate the severity of the immuno-inflammatory state in COVID-19, lactate dehydrogenase indicates the tissue damage that has occurred [23]. Hence, the five parameters are individually associated with mortality, and the final model had good predictive accuracy. Moreover, the laboratory parameters included in the model are routinely available in the ICU. 

The limitations of our study are its retrospective nature, small sample size, and absence of external validation. Nevertheless, we included all relevant parameters while limiting their number to a minimum. Hence, the model accuracy is unlikely to change with a higher number of patients. The model was also limited to studying mortality as a binary outcome and did not take into account the cause of mortality or other ICU complications. 

## Conclusions

Mortality in our study of ICU COVID-19 patients was 40.8%. A model incorporating CCI, SOFA score, D-dimer, LDH, and N/L ratio could predict mortality with good accuracy. Moreover, each of these parameters was independently associated with mortality. This prediction model, with routinely available parameters, can help in targeted therapy for ICU patients more effectively. 
